# Two Patients with Extremely Elevated Tumor Markers: Where Is the Malignancy?

**DOI:** 10.1155/2011/123743

**Published:** 2011-06-16

**Authors:** Patrick P. J. van der Veek, Wouter H. de Vos tot Nederveen Cappel, Alexandra M. J. Langers, Bart van Hoek

**Affiliations:** ^1^Departments of Internal Medicine, Gastroenterology, and Hepatology, Medisch Centrum Haaglanden, P.O. Box 432, 2501 CK The Hague, The Netherlands; ^2^Department of Gastroenterology and Hepatology, Isala Clinics, P.O. Box 10500, 8000 GM Zwolle, The Netherlands; ^3^Department of Gastroenterology and Hepatology, Leiden University Medical Center, 2300 RC Leiden, The Netherlands

## Abstract

Serum tumor markers are useful to evaluate a cancer's response to treatment, for early detection of cancer relapse, and, in some cases, to diagnose malignancy. In this paper, we present two patients with significantly elevated serum tumor markers *without* evidence of malignant disease. An 18-year-old patient suffering from autoimmune hepatitis had markedly increased alpha-fetoprotein (aFP) levels (2,002 *μ*g/L; normal <10 ug/L). Extensive imaging showed no signs of hepatocellular carcinoma or other cancer, and treatment with Prednisone led to rapid normalization of both liver enzymes and aFP. The second patient, a 60-year-old female with painless jaundice due to biliary stone disease, had very high serum levels of CA19-9 (18,000 kU/L, normal <27 kU/L). Liver biochemistry and serum CA19-9 concentration decreased to almost normal values (45 kU/L) after biliary stenting. These cases demonstrate that serum tumor markers can be elevated in benign disease and are therefore not appropriate to diagnose cancer.

## 1. Introduction

Serum tumor markers are useful to evaluate a cancer's response to treatment, for early detection of cancer relapse, and, in some cases, to diagnose malignancy. Here, we present two patients with significantly increased serum tumor markers without evidence of malignant disease.

## 2. Case A

**Figure 1:**
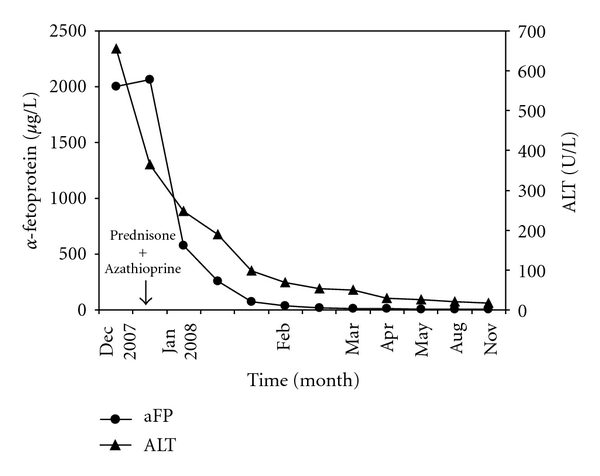
Serum alpha-fetoprotein (aFP) and alanine aminotransferase (ALT) concentrations in an 18-year-old male patient with autoimmune hepatitis. Both markers normalized in several months after treatment with Prednisone and Azathioprine was started.

An 18-year-old male patient was admitted for progressive jaundice, which had started after an episode of binge drinking during a holiday in Italy 5 months before presentation. Jaundice had been intermittently present since but had worsened during the last few weeks. He suffered from unusual fatigue and nonspecific upper abdominal pain that was not related to meal ingestion. Urine and stools were normal. On admission, he had stopped drinking alcohol for several months and denied any drug abuse. Physical examination showed a mild jaundice but was otherwise unremarkable. Laboratory tests revealed a mild macrocytic anemia (hemoglobin 7.3 mmol/L (normal (N) 8.5–11.0 mmol/L), MCV 101 fl (N 80–100 fl), and marked elevation of liver biochemistry (total bilirubin 100 *μ*mol/L (N 0–17 *μ*mol/L), conjugated bilirubin 64 *μ*mol/L (N 0–5 *μ*mol/L), alkaline phosphatase (ALP) 155 U/L (N 40–120 U/L), gamma-GT (gGT) 254 U/L (N 5–55 U/L), aspartate aminotransferase (AST) 1,025 U/L (N 5–35 U/L), alanine aminotransferase (ALT) 656 U/l (N 5–45 U/L)). Antibodies against smooth muscle (ASMA) and nuclei (ANA) were positive, and gamma globulin level was elevated (IgG, 25.1 g/L; N 7.0–16.0 g/L)), suggesting autoimmune hepatitis. Anti-DNA was negative. Remarkably, alpha-fetoprotein (aFP) concentration, which was routinely measured in the standard workup for patients with liver enzyme abnormalities, was also significantly increased to 2,002 *μ*g/L (upper limit of normal (ULN) 10 *μ*g/L). Test results for hepatitis A, B, and C, Epstein-Barr virus, Cytomegalovirus, and antimitochondrial antibodies were all negative. No evidence was found for Wilson's disease, hemochromatosis; alfa-1-antitrypsine genotype was partly deficient (PI-MS) but quantitative analysis was normal. Urinalysis was also completely normal. Abdominal ultrasound showed normal liver parenchyma without focal liver lesions and no signs of portal hypertension. Because of the extraordinary aFP increase, an additional abdominal CT scan was performed to exclude hepatocellular carcinoma (HCC), seminoma, and nonseminomatous germ cell tumor, all of which could not be demonstrated. Liver biopsy showed a periportal and lobular inflammatory infiltrate, piecemeal necrosis, abundance of plasma cells, and extensive collapse of liver parenchyma confirming the diagnosis of autoimmune hepatitis. Immediately after biopsy, Prednisone 40 mg once daily was started, which led to rapid improvement and, eventually, normalization of liver biochemistry. Interestingly, aFP levels also decreased rapidly at the same rate as ALT (Figure 1). Prednisone was slowly tapered with synchronous introduction of Azathioprine. At present, complete remission is maintained with Prednisone 2,5 mg and Azathioprine 150 mg once daily.

## 3. Case B

**Figure 2:**
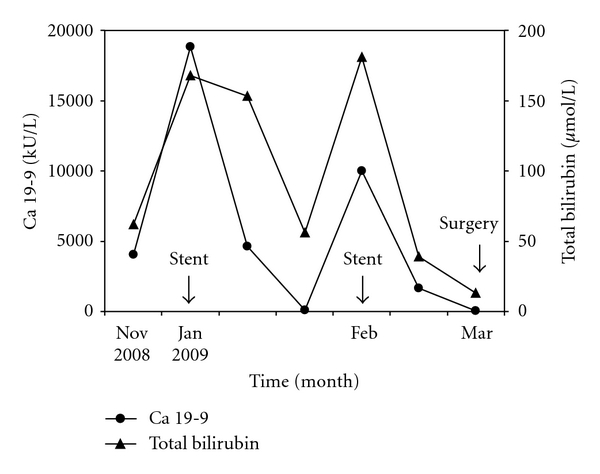
Serum Cancer Antigen 19-9 (CA 19-9) and total bilirubin concentrations in a 60-year-old female patient with obstructive jaundice due to a large biliary stone. Both markers rapidly decreased after endoscopic placement of a biliary endosprothesis. Surgery was performed in March 2009.

A 60-year-old female patient was admitted to a regional hospital for painless jaundice, pruritus, and weight loss after a period of epigastric pain. Physical examination showed no abnormalities other than jaundice. Liver biochemistry was consistent with obstructive jaundice (total bilirubin 62 *μ*mol/L, conjugated bilirubin 41 *μ*mol/L, ALP 549 U/L, gGT 950 U/L, AST 74 U/L, ALT 332 U/L) but also showed extreme elevation of Cancer Antigen (CA) 19-9 (18,000 kU/L, ULN 27 kU/L). Abdominal ultrasound showed multiple stones in the gallbladder and a 13 mm stone in the distal cystic duct; there were no signs of cholecystitis. Both the common hepatic duct and intrahepatic ducts were dilated. During admission, liver enzymes improved spontaneously and the patient was discharged, awaiting cholecystectomy. However, she was readmitted with a relapse of painless jaundice before surgery had been performed. Abdominal CT scan now showed a dilated CBD and intrahepatic ducts, but no signs of malignancy. MRCP revealed a 13 mm distal CBD stone. The patient was transferred to our hospital when she developed cholangitis. At ERCP, stone removal from the CBD was impossible due to the size of the gallstone, and a biliary endoprosthesis was placed. Serum CA19-9 concentration decreased to almost normal values (45 kU/L) after biliary stenting. One month later she was readmitted for relapse of cholangitis after endoprothesis luxation. Again, CA19-9 was >10,000 kU/L and returned to almost normal (45 kU/L) after stent placement. A second attempt for endoscopic crushing and stone extraction was not successful, and she eventually underwent cholecystectomy with choledochotomy and stone removal. No signs of malignancy were detected during surgery. The histopathology of the removed gallbladder showed cholecystolithiasis and signs of chronic inflammation. Serum levels of bilirubine, and CA19-9 are shown in Figure 2.

## 4. Discussion

Alpha-fetoprotein is a 68-kilodalton polypeptide that is produced in the fetal liver and yolk sac. Serum levels of aFP are undetectable in healthy individuals but can be increased in a number of conditions, including hepatocellular carcinoma (HCC), seminoma and nonseminomatous germ cell tumors, and gastric, biliary, and pancreatic cancers. Levels of aFP can also be slightly elevated (up to 500 *μ*g/L) in pregnancy, reflecting fetal production, or sometimes higher in several obstetric complications. Increased levels can also be present in chronic viral hepatitis. For instance, up to 17% of patients with chronic hepatitis C infection and advanced fibrosis or cirrhosis without evidence for HCC have modest elevation of serum aFP concentrations, which improve upon treatment with pegylated interferon alpha-2a and ribavirin [1]. In general, serum concentrations >400 *μ*g/L in high-risk patients (cirrhosis, chronic hepatitis B infection) in the presence of a focal lesion of more than 2 cm with arterial hypervascularization on one imaging modality are considered diagnostic for HCC [2]. Lower levels in patients with HCC are common, and normal serum aFP concentrations can be found in a significant number of patients with HCC [3]. False positive results may occur in alcohol and drug abuse and other states of chronic liver damage, but values are usually <100 *μ*g/L.

Development of HCC in preexisting autoimmune hepatitis can cause serum aFP elevation as was reported previously [4–7]. Cirrhosis was present in almost all of these patients [4–6] or not reported [7]. In our case, however, liver biopsy did not demonstrate fibrosis or cirrhosis, and abdominal ultrasound and CT scan showed no underlying malignancy as an explanation for the rise in aFP. Non-malignant serum aFP elevation may result from altered hepatocyte-hepatocyte interaction and loss of normal architectural arrangement, such as seen in fibrosis and cirrhosis [8] or, as in our case, widespread liver tissue collapse. In contrast, experiments in regenerating mouse liver tissue after CCI4 poisoning demonstrated that aFP-producing cells were normally differentiated hepatocytes without any structural signs of damage [9]. Thus, liver tissue regeneration during hepatic inflammation may be an alternative explanation for the rise in serum aFP in our patient with autoimmune hepatitis.

To our knowledge, this paper is the first to describe a patient with autoimmune hepatitis and this extreme elevation of serum aFP up to levels suggestive of HCC (>400 *μ*g/L) in whom no malignancy was found. Both liver biochemistry and aFP normalized following treatment with high-dose glucocorticoids. A recent report described a 59-year-old patient with a synchronous diagnosis of systemic lupus erythematosus (SLE) and autoimmune hepatitis in whom aFP levels were modestly elevated (320 *μ*g/L) [10]. In this patient, levels of aFP returned to normal within weeks after treatment with Prednisone 30 mg/day and Azathioprine 100 mg/day was started. Our patient had no signs of other underlying autoimmune diseases. 

CA19-9, a tumor-associated antigen, is recognized by the monoclonal antibody 116NS-19-9. The antigenic determinant on CA19-9 that is recognized by 116NS-19-9 is a sialylated lacto-N-fucopentaose II, the so-called sialyl Lewis antigen. CA19-9 is used as a tumor marker of cancers in the gastrointestinal tract, such as pancreatic and biliary tract cancers, and colon, esophageal, and hepatic carcinomas. CA19-9 levels may also be elevated in several benign conditions in individuals with the Lewis antigen positive phenotype. These include inflammation or proliferation of noncancerous tissue, probably due to hypersecretion by normal epithelial cells (i.e., pancreatitis, pancreatic cysts, cholangitis, bronchiectasis, and pulmonary fibrosis) and obstruction of CA19-9 discharge pathways. The correlation between serum CA19-9 levels and serum cholestasis parameters (ALP, bilirubin) is well established, and CA19-9 elevation in biliary obstruction is probably caused by leakage of biliary mucins into the serum (i.e., pancreatic or biliary duct stenosis due to gallstones). Malfunction in organs that metabolize CA19-9 (chronic hepatitis, chronic glomerulonephritis) may also lead to elevated serum levels of CA19-9 [11]. Generally, levels greater than 1,000 kU/L are rare in benign conditions, although one study found CA19-9 levels of >1,000 U/ml to be present in 4.7% of patients with cholangitis or cholestasis secondary to benign disease [11–13].

On initial presentation, our patient probably had obstructive jaundice due to Mirizzi's syndrome. CA19-9 was routinely measured in the workup for painless jaundice and was extremely elevated to a maximum of 18,000 kU/L. Only one previous case report demonstrated highly increased levels of CA19-9 in a patient with Mirizzi's syndrome [14]. The clear correlation between serum concentrations of CA19-9, ALP and bilirubin, with normalization after biliary stent placement in the absence of tumor on CT-scan and ERCP, strongly suggests that the elevation of CA19-9 was due to cholestasis and not caused by malignant disease.

In conclusion, these two cases not only demonstrate that tumor markers can be elevated in benign disease but also emphasize that elevated serum tumor markers alone are not suitable for establishing a diagnosis of malignancy.

## Abbreviations

aFP: Alpha-fetoprotein
ALT: Alanine aminotransferase
ALP: Alkaline phosphatase
AST: Aspartate aminotransferase
CA 19-9: Cancer Antigen 19-9
CBD: Common bile duct
HCC: Hepatocellular carcinoma
N: Normal values
ULN: Upper limit of normal.
